# Effect of pre-treatment with oral short-acting contraceptives on assisted reproductive technology outcomes in patients with polycystic ovary syndrome: a meta-analysis

**DOI:** 10.3389/fendo.2025.1545508

**Published:** 2025-06-05

**Authors:** Yufei Liang, Qiquan Zhang, Zhaoxia Lou

**Affiliations:** Department of Gynecology and Obstetrics, Huzhou Maternity & Child Health Care Hospital, Huzhou, Zhejiang, China

**Keywords:** polycystic ovary syndrome, combined oral contraceptives, assisted reproductive technology, live birth rate, meta-analysis

## Abstract

**Objective:**

This study aims to investigate the effects of pre-treatment with Combined Oral Contraceptives (COC) on the prognosis of individuals with polycystic ovary syndrome (PCOS) who undergo assisted reproductive technology (ART).

**Methods:**

Three databases (PubMed, Embase, and Cochrane Library) were searched from their establishment until February 23, 2024. Literature screening was performed based on predefined inclusion and exclusion criteria. Meta-analysis was executed using Stata 14.0 software, with outcomes expressed as mean differences (MD) and odds ratios (ORs), with 95% confidence intervals (CIs).

**Results:**

Our comprehensive analysis comprised of 11 studies, encompassing a total of 4413 patients diagnosed with PCOS. Meta-analysis results revealed that, compared to no pre-treatment, the use of COC pre-treatment did not significantly improve clinical pregnancy rates (OR: 0.96, 95% CI: 0.85 to 1.09), cumulative pregnancy rates (OR: 1.13, 95% CI: 0.92 to 1.39), or implantation rates (OR: 1.16, 95% CI: 0.99 to 1.36). However, the use of COC pre-treatment was found to be linked to a higher rate of miscarriage (OR: 1.29, 95% CI: 1.01 to 1.65). Additionally, it did not have a significant impact on the rate of live births (OR: 0.81, 95% CI: 0.68 to 0.97), cumulative live births (OR: 0.90, 95% CI: 0.78 to 1.04), or the occurrence of OHSS (OR: 0.83, 95% CI: 0.54 to 1.28). Gonadotropin dosage required for ovarian stimulation also showed no significant difference (MD: -15.32, 95% CI: -79.79 to 49.15). At the same time, we analyzed different ovulation induction regimens and found that there was no statistically significant miscarriage rate between the GnRH agonist (standard long regimen) after COC pre-treatment and the control group (OR: 1.12, 95% CI: 0.79 to 1.59), while there was no significant difference between the live birth rate with GnRH agonist (standard long regimen) after contraceptive pre-treatment and the control group (OR: 0.85, 95% CI: 0.64 to 1.14).

**Conclusions:**

Administering COC pre-treatment for patients with PCOS undergoing ART does not provide substantial advantages in terms of clinical pregnancy, cumulative pregnancy, live birth rates, or the reduction of OHSS incidence. However, it is associated with an increased risk of miscarriage.

**Systematic Review Registration:**

https://www.crd.york.ac.uk/prospero, identifier CRD42024528652.

## 1 Introduction

Polycystic Ovary Syndrome (PCOS) is a common and intricate hormonal disorder that primarily affects women. It is widely acknowledged as a significant issue in public health (1). The incidence of PCOS varies globally, with reported rates ranging from 4% to 21% among women of reproductive age, depending on the diagnostic criteria employed and the populations studied (2, 3). Diagnostic standards for PCOS encompass clinical or biochemical evidence of hyperandrogenism, oligo- or anovulation, and the presence of polycystic ovaries on ultrasound, underscoring the critical role of androgen excess in the pathophysiology of the condition (4, 5). Moreover, the connection between the syndrome and anovulation establishes it as a key determinant of infertility in women of reproductive age (6).

Management of PCOS-related infertility involves a spectrum of interventions, starting with ovulation induction using clomiphene citrate or letrozole, and potentially escalating to assisted reproductive technologies (ART) such as *in vitro* fertilization (IVF), especially in cases where initial treatments fail or are contraindicated due to male factor infertility, tubal blockage, or an exaggerated response to ovulatory drug (7, 8). Controlled ovarian stimulation, oocyte retrieval, and *in vitro* fertilization are integral components of ART; however, women with PCOS face distinct challenges in this context, including a heightened risk of ovarian hyperstimulation syndrome (OHSS) due to their pronounced response to stimulation (9). Although women with PCOS often produce many retrieved oocytes, they may have a lower-than-expected proportion of oocytes that are fully mature (10), further complicated by the irregular menstrual cycles’ characteristic of anovulation.

To ameliorate these issues, pre-treatment strategies, including lifestyle interventions, metformin administration, progestin therapy, and the use of Combined Oral Contraceptives (COC), have been employed (11). Among these options, COC is particularly favored because it simultaneously suppresses elevated luteinizing hormone (LH) and androgen levels, regulates menstrual cycles, improves synchronization of follicular development, and enhances cycle programming prior to ART. Compared with estrogen-only or progestin-only regimens, COCs provide dual suppression of gonadotropins and hyperandrogenism, which is crucial for mitigating premature LH surges, reducing androgen-related follicular dysfunction, and minimizing the risk of ovarian hyperstimulation in PCOS patients (12). These advantages make COC pre-treatment a clinically preferred strategy in ART protocols for PCOS patients. COC pre-treatment, commonly used to regulate menstrual cycles and reduce hyperandrogenism, are also applied as a pre-treatment in PCOS patients undergoing ovulation induction. However, existing literature presents divergent outcomes regarding the impact of COC pre-treatment on ART outcomes in PCOS patients, including clinical pregnancy rates, incidence of OHSS, and miscarriage rates (13, 14).

This study intends to systematically review and analyze the impact of COC pre-treatment on the clinical outcomes of ART in women with PCOS. This study aims to comprehensively assess the efficacy and safety of COC pre-treatment in a specific patient population by examining various ART protocols.

## 2 Methods

This systematic review and network meta-analysis were conducted in strict accordance with the Preferred Reporting Items for Systematic Reviews and Meta-Analyses (PRISMA) guidelines (15). and the protocol of this paper was registered in PROSPERO (registration number CRD42024528652). Ethics approval was not required.

### 2.1 Search strategy

A comprehensive literature search was performed across several databases, including PubMed, Embase, and Cochrane Library. The search covered all records from the inception of each database up to January 6, 2025, without language restrictions. The search strategy combined Medical Subject Headings (MeSH) and free-text terms related to”Polycystic Ovary Syndrome” (PCOS) and “Combined Oral Contraceptives (COC).” The detailed search terms and full search strategy are provided in the **Supplementary Material**. To ensure a comprehensive identification of pertinent studies, we also manually examined the reference lists of included articles and relevant reviews. Screening of titles and abstracts followed by full-text evaluation was independently conducted by two researchers (Yufei Liang and Zhaoxia Lou), using a pre-defined inclusion and exclusion criteria to determine study eligibility. Any inconsistencies among reviewers during any phase of the selection process were handled through deliberation or, in case of failure to establish a consensus, by intervention from a third reviewer (Qiquan Zhang).

### 2.2 Study selection

The selection criteria for this study were carefully and precisely established to guarantee a concentrated and pertinent analysis :(1)We included studies involving patients diagnosed with PCOS according to the Rotterdam criteria;(2)who were undergoing ART treatments, specifically IVF or intracytoplasmic sperm injection (ICSI), where gonadotropin was used to control ovarian stimulation;(3) Only parallel-design studies that directly compared the outcomes of ART with and without the pre-treatment of COC were taken into account ;(4) the type of study is a controlled clinical study; (5) The relevant indicators of the study comparison included clinical pregnancy rate, cumulative pregnancy rate, implantation rate, miscarriage rate, live birth rate, cumulative live birth rate, OHSS incidence, and gonadotropin dose.

Exclusion criteria were strictly applied to isolate the effect of COC pre-treatment on ART outcomes.:(1)Studies were excluded if they did not utilize gonadotropin for controlled ovarian stimulation, involved women undergoing timed intercourse or intrauterine insemination instead of ART,;(2)used oral contraceptive pre-treatments other than COC, such as estrogen-only or progestin-only medications;(3)did not assess pregnancy outcomes;(4)did not independently report on the effects of COC pre-treatment;(5)Studies involving *in-vitro* maturation (IVM) were eliminated to retain focus on the influence of COC pre-treatment within typical ART protocols;(6) Reviews, animal tests, and case reports were excluded.

### 2.3 Data extraction

Upon the systematic retrieval and organization of articles in EndNote X9, two authors(Yufei Liang and Zhaoxia Lou) independently extracted data from studies that met our inclusion criteria. This process involved collecting key information such as publication details (author, title, year, journal), participant demographics (age, country), interventions, and outcomes measured. Discrepancies between reviewers were resolved through consensus, ensuring data accuracy and reliability. For quantitative analysis, standard deviation (SD) was calculated from reported standard errors (SE) in experimental and control groups using the formula: SD = SE × √n. In cases lacking SE or SD, we estimated SD from available statistical measures like confidence intervals, t-values, quartiles, ranges, or p-values, following the Cochrane Handbook (section 7.7.3). This method facilitated accurate data comparison across numerous studies. We will contact the first and/or corresponding authors to gather any additional information required.

### 2.4 Outcomes

The results of this analysis focused on clinical ART outcomes, specifically the clinical pregnancy rate, cumulative pregnancy rate, miscarriage rate, live birth rate, cumulative live birth rate, OHSS, gonadotropin dosage, and implantation rate.

### 2.5 Risk of bias

The assessment of the risk of bias within the included studies was conducted with rigorous precision by two independent reviewers. For randomized controlled trials (RCTs), we utilized the Revised Cochrane Risk of Bias Tool for Randomized Trials (RoB 2) (16), which evaluates randomization, deviations from intended interventions, missing outcome data, outcome measurement, and selective reporting. For all non-randomized studies, we replaced previous instruments with the Risk of Bias in Non-randomized Studies of Interventions (ROBINS-I) tool (17). ROBINS-I appraises seven domains— (1) confounding, (2) participant selection, (3) classification of interventions, (4) deviations from intended interventions, (5) missing data, (6) outcome measurement, and (7) selection of the reported result—and assigns an overall judgement of “low,” “moderate,” “serious,” or “critical” risk of bias (18). All conflicts among reviewers were resolved through discussion or, if necessary, arbitration by a third reviewer to ensure consistency.

### 2.6 Data synthesis

This study utilized a methodical meta-analytical approach to investigate the varying effects of COC pre-treatment on the outcomes of ART in patients with PCOS. Additionally, a thorough subgroup analysis was conducted to examine the relationship between contraceptive pre-treatment and different ART protocols. Specifically, subgroup analyses were stratified according to three comparison cohorts: COC pre-treatment followed by a GnRH antagonist protocol (CAA) versus a GnRH antagonist protocol without pre-treatment (AA); COC pre-treatment followed by a GnRH agonist protocol (standard long protocol, CAL) versus a GnRH agonist protocol without pre-treatment (AL); and comparisons between CAL and CAA.

A series of pairwise meta-analyses were performed to compare the outcomes of the experimental and control groups. Heterogeneity among the included studies was quantitatively assessed using the I² statistic, where values of I² < 25%, 25 - <50%, 50 - <75%, and ≥75% were indicative of incredibly low, low, moderate, and high degrees of heterogeneity, respectively (19). Based on the extent of heterogeneity observed, data were synthesized using either a fixed-effect model (when the I² statistic p > 0.1) or a random-effects model (when the I² statistic p ≤ 0.1) to aggregate study results. To make sure that our results were strong, we did sensitivity analyses using two different methods: (1) replacing the fixed-effect model with a random-effects model, and (2) taking out one primary study at a time from the pooled analysis to see what effect that had on the overall outcome (20).

All statistical analyses were performed utilizing STATA software version 14.0 (Stata Corp LP, College Station, TX, USA). Effect measures were expressed as odds ratios (ORs) or mean differences (MDs) with corresponding 95% confidence intervals (CIs). For all tests, a two-tailed p-value of ≤ 0.05 was considered statistically significant.

## 3 Results

### 3.1 Characteristics of included studies

**Figure 1:**
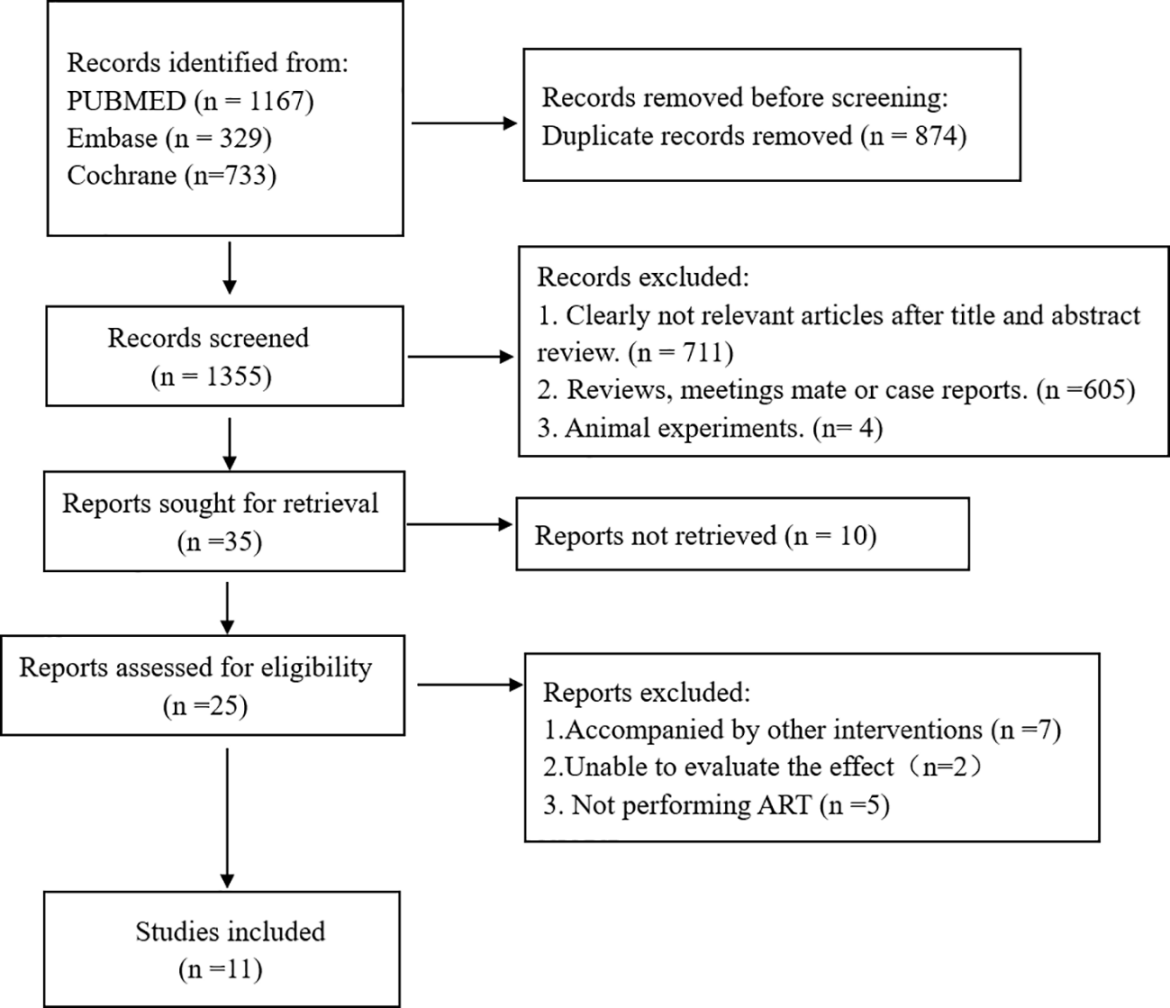
A search flow chart detailing the search process.

2229 records were identified during the initial electronic searches. Nevertheless, the analysis was conducted using a mere 11 records, as depicted in **Figure 1**. These 11 records consist of 2 RCTs (21, 22), 2 prospective cohort studies (23, 24), and 7 retrospective cohort studies (12, 25–30). **Table 1** outlines the attributes of the studies included. Subgroup analyses were used to examine the results reported by both RCTs and cohort studies. The COC group consisted of 2642 participants, while the control group had 1771 participants. The included studies were conducted in different countries: six in China, two in Turkey, and one each in Iran, Korea, and France (**Table 1**).

**Table 1:** Characteristics and quality score of included studies.

Study,year	Country	Study type	Participants(n)	Age (mean+/-SD) Or Median [IQR]	COC type dose	Pituitary suppression	ART	ET	Results
Wei (23) (2017)	China	Prospective cohort	COC(902)	28.0+/-3.0	EE 30 µg+desogestrel 0.15 mg EE35µg+cyproterone acetate 2 mg EE 30 µg+drospirenone 3 mg	GnRH antagonist	IVF-ET	Fresh and FET	COC treatment was associated with lower rate of live birth
			Control(323)	28.4+/-3.0					
Pan (13) (2014)	China	Retrospective cohort	COC(208)	/	unspecified	GnRHa long, GnRHa short and GnRH antagonist (GnRHant) protocols as the supplemental	IVF-ET or ICSI	Fresh and FET	COC treatment significantly improved the implantation and pregnancy rates
			Control(292)	/					
Kalem (25) (2017)	Turkey	Retrospective cohort	COC(292)	30.00(7)	EE 30μg and gestodene 0.075mg	GnRH antagonist	IVF-ET or ICSI	Fresh	No difference in clinical outcomes
			Control(376)	29.00(8)					
Ozmen (26) (2014)	Turkey	Retrospective cohort	COC(143)	28.0+/-5.1	EE30mg+ 3mg drospirenone	GnRH antagonist	ICSI	Fresh	No benefcial or adverse outcomes with COC treatment
			Control(59)	27.6+/-4.3					
Wu (27) (2012)	China	Retrospective cohort	COC(53)	30.71+/-4.42	EE35µg+cyproterone acetate 2 mg	GnRHa long	IVF-ET or ICSI	Fresh	Improvement in percentage of mature oocytes No difference in clinical outcomes
			Control(32)	30.92+/-3.5					
Xu (28) (2019)	China	Retrospective cohort	COC(779)	/	EE30µg+desogestrol 0.15mg EE35µg+cyproterone acetae 2mg EE30µg+drospirenone 3mg	GnRHa long	IVF-ET	Fresh and FET	COC treatment was not directly responsible for live birth rate reduction
			Control(246)	/					
Decanter (24) (2013)	France	Prospective cohort	COC(45)	28[22.0-35.6]	EE30μg and desogestrel 150μg	GnRHa long	IVF-ET or ICSI	Fresh	Clinical and ongoing pregnancy rates were significantly lower in the OCP
			Control(57)	30[23.9-35.0]					
Tehraninejad (21) (2010)	Iran	RCT	COC(45)	28.99+/-6.1	EE30µg+drospirenone 3mg	GnRHa long	IVF-ET or ICSI	Fresh	no statistically of clinical pregnancy rates
			Control(45)	30.43+/-5.08		GnRH antagonist			
Shin (22) (2018)	South Korea	RCT	COC ^1^(24)	32.72+/-2.68	EE30µg+cyproterone acetae 2mg	GnRH antagonist	IVF-ET or ICSI	Fresh	Early antagonist reduced OHSS
			COC ^2^(11)	33+/-2.25					
				GnRHa long					
Liu (29) (2023)	China	Retrospective cohort	COC(119)	31.0+/-3.3	EE35µg+cyproterone acetae 2mg EE30µg+drospirenone 3mg	GnRH antagonist	IVF-ET or ICSI	Fresh and FET	COC treatment signifcantly improved the Cumulative pregnancy rate
			Control(106)	31.2+/-3.3					
Zhou (30) (2023)	China	Retrospective cohort	COC(21)	31.52 + 2.56	unspecified	GnRH antagonist	IVF-ET or ICSI	Fresh	Cumulative pregnancy rate,Cumulative live birth rate have Cumulative live birth rate
			Control(235)	30.57+/-4.18					

COC, combined oral contraceptives;, ART, assisted reproductive technology; ET, embryo transfer; IVM, *in vitro* maturation; ICSI, intracytoplasmic sperm injection; RCT, randomized controlled trial; GnRHa, gonadotropinreleasing hormone analogue; IVF-ET, *in vitro* fertilization and embryo transfer; EE, ethinyl estradiol; GnRH, gonadotropin-releasing hormone; FET, frozen embryo transfer.

The reporting of women’s age across studies varied, employing several methods such as mean ± SD per group, median and range for all women included, or range for all subjects. Therefore, it was not possible to directly compare age across these studies in this meta-analysis. Regarding ovulation induction protocols, three studies implemented Gonadotropin-Releasing Hormone (GnRH) agonist protocols, while five studies utilized GnRH antagonist protocols. One study employed varied protocols, including a long GnRH agonist regimen, a short GnRHa course, and supplementation with a GnRH antagonist. Two studies each applied GnRH antagonist and agonist protocols, respectively.

### 3.2 Risk of bias

Upon reassessing the included studies with updated tools, the risk of bias was evaluated using the RoB 2 tool for RCTs and the ROBINS-I tool for non-randomized studies. For the two RCTs, both studies were judged as having a low risk of bias across all domains, and their overall bias judgment was low. For the nine non-randomized studies, the overall risk of bias was moderate in seven studies and serious in two studies. Specifically, in the domain of bias due to confounding, seven studies were assessed as moderate risk and two studies as serious risk; for bias in selection of participants, bias due to missing data, and bias in selection of the reported result, seven studies were judged as low risk and two studies as moderate risk; for bias in classification of interventions and bias in measurement of outcomes, all nine studies were rated as low risk. Detailed domain-level assessments are presented in **Supplementary File 2** (**Supplementary Tables 2.1, 2.2**).

### 3.3 The results of meta-analysis

#### 3.3.1 Implantation rate

**Figure 2:**
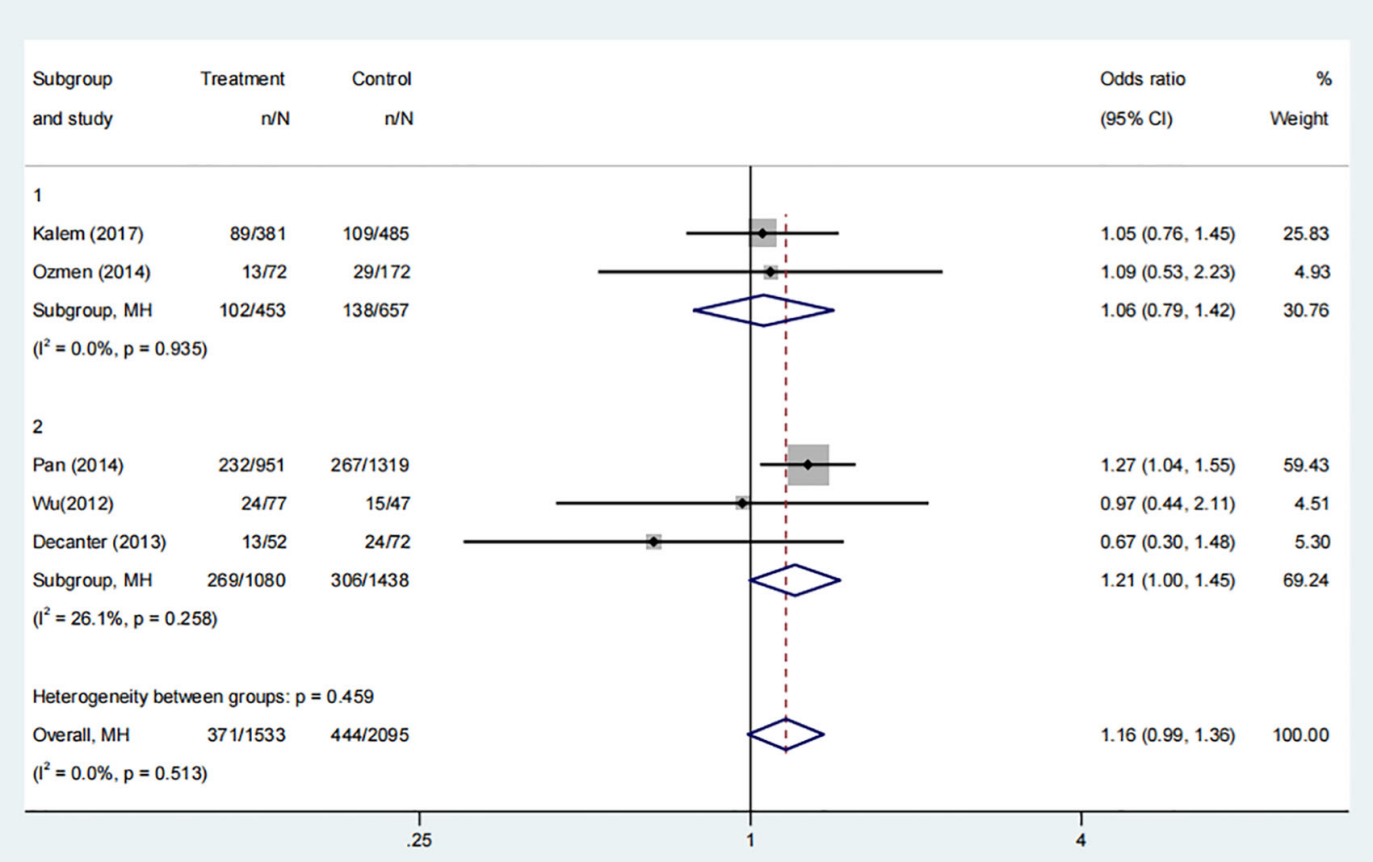
Analysis of the Implantation rate,Including the comparison between different ovulation induction regimens.

Five studies, involving 2,813 participants, examined the implantation rate in groups that received COC pre-treatment compared to control groups. The meta-analysis found no significant difference in implantation rates between groups treated with COC pre-treatment and those without, as shown in **Figure 2** (OR: 1.16, 95% CI: 0.99 to 1.36, P>0.05). The subgroup analysis of the two COC pre-treatment protocols, CAA, and CAL indicated that there was no statistically significant difference in implantation rates compared to the control groups that did not undergo COC pre-treatment. This suggests that COC pre-treatment does not have a significant impact on the likelihood of implantation in ART cycles.

#### 3.3.2 Clinical pregnancy rate

**Figure 3:**
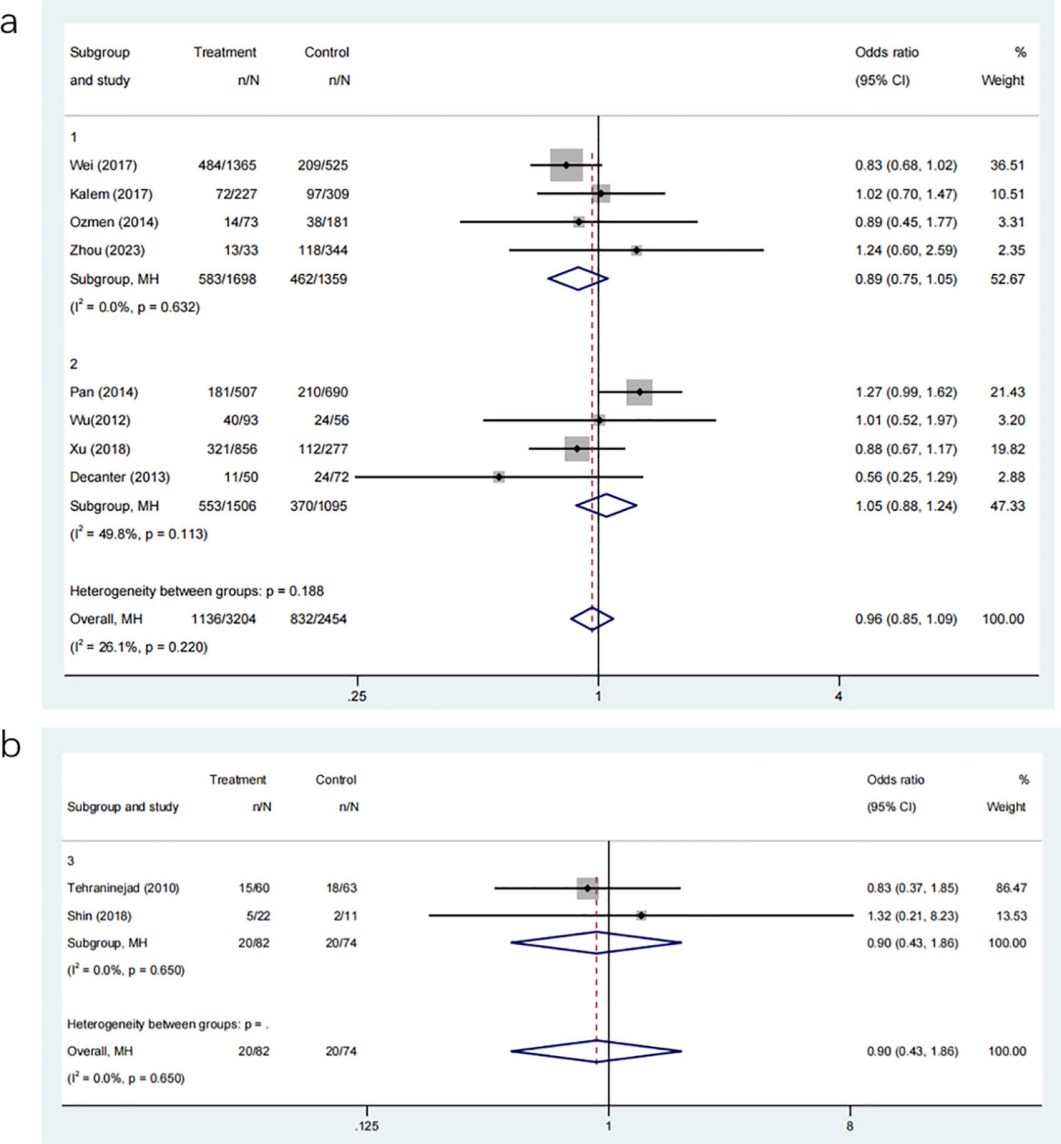
**(a)** Analysis of the clinical pregnancy rate,including the comparison between different ovulation induction regimens; **(b)** Analysis of clinical pregnancy rate after COC preconditioning with different ovulation promoting schemes.

The meta-analysis we conducted consisted of eight studies, involving 3690 participants. The objective was to compare the clinical pregnancy rates between groups that received COC pre-treatment and groups that did not receive any pre-treatment. The pooled results indicated no significant advantage in clinical pregnancy rates for the COC pre-treatment group compared to the control group, as shown in **Figure 3a** (OR: 0.96, 95% CI: 0.85 to 1.09). Comparing the different COC pre-treatment protocols, specifically CAA (**Figure 3a**: OR: 0.89, 95% CI: 0.75 to 1.05) and CAL (**Figure 3a**: OR: 1.05, 95% CI: 0.88 to 1.24), the control groups also did not show any significant differences. CI: 0.75 to 1.05) and CAL (**Figure 3a**: OR: 1.05, 95% CI: 0.88 to 1.24), also showed no significant differences compared to the control groups.

Two studies, involving 116 participants, compared the clinical pregnancy rates between the CAA and CAL COC pre-treatment protocols. The analysis showed no significant difference between the two pre-treatment approaches (**Figure 3b**: OR: 0.90, 95% CI: 0.43 to 1.86).

#### 3.3.3 Cumulative pregnancy rate

**Figure 4:**
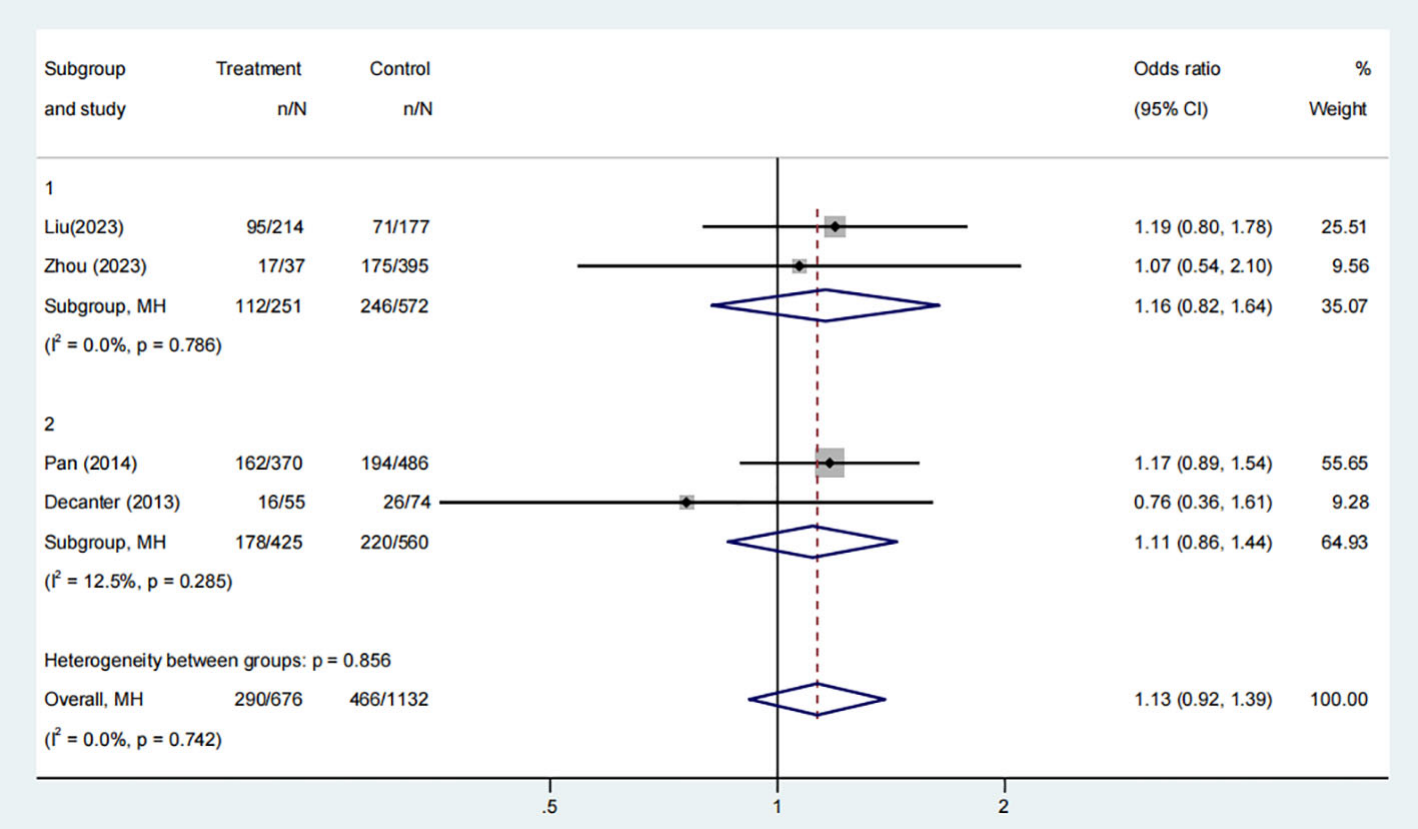
Analysis of cumulative pregnancy rate,Including the comparison between different ovulation induction regimens.

Four studies, comprising 1052 participants, evaluated the combined pregnancy rates of COC pre-treatment groups compared to control groups. The meta-analysis revealed no significant difference in cumulative pregnancy rates between the groups (**Figure 4**, OR: 1.13, 95% CI: 0.92 to 1.39). Subgroup analysis of the two COC pre-treatment protocols, CAA (**Figure 4**, OR: 1.16, 95% CI: 0.82 to 1.64) and CAL (**Figure 4**, OR: 1.11, 95% CI: 0.86 to 1.44), also found no significant differences when compared with control groups without pre-treatment.

#### 3.3.4 Miscarriage rate

**Figure 5:**
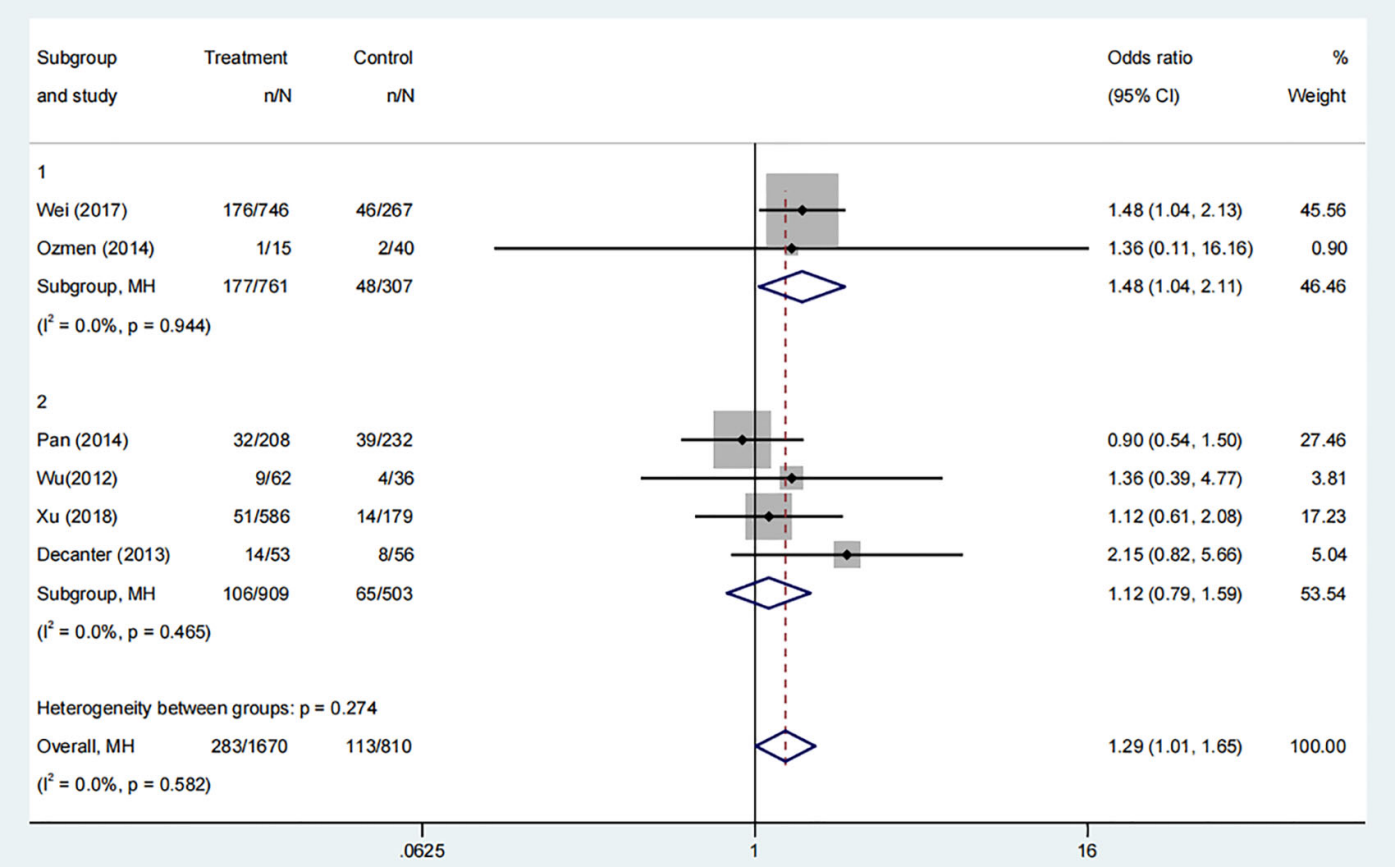
Analysis of miscarriage rate,Including the comparison between different ovulation induction regimens.

Six studies, comprising 1435 participants, compared the miscarriage rates between COC pre-treatment groups and control groups. The analysis indicated a higher miscarriage rate in the COC pre-treatment group compared to the control group (**Figure 5**, OR: 1.29, 95% CI: 1.01 to 1.65). Subgroup analysis showed that the CAA pre-treatment group had a higher risk of miscarriage than the control group (**Figure 5**, OR: 1.48, 95% CI: 1.04 to 2.11), while the CAL pre-treatment group showed no significant difference (**Figure 5**, OR: 1.12, 95% CI: 0.79 to 1.59).

#### 3.3.5 Live birth rate

**Figure 6:**
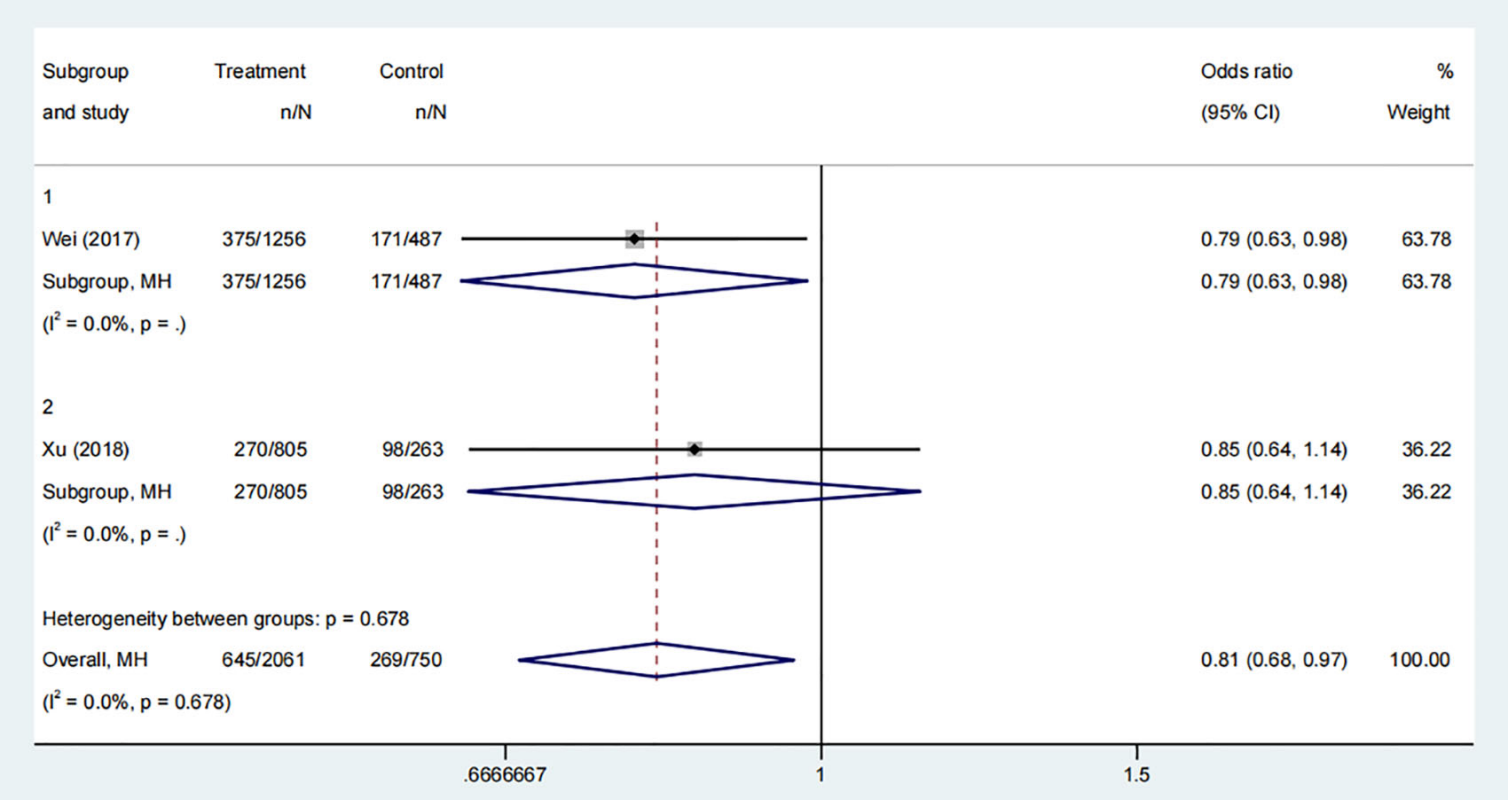
Analysis of live birth rate, including the comparison between different ovulation induction regimens.

Two studies, involving 1897 participants, compared the live birth rates of groups who received COC pre-treatment with those of control groups. The meta-analysis revealed a statistically significant increase in the live birth rate in the control group compared to the COC pre-treatment group (**Figure 6**, Odds Ratio: 0.81, 95% Credible Interval: 0.68 to 0.97). The subgroup analysis revealed that the control group had a higher live birth rate compared to the CAA pre-treatment protocol (**Figure 6**, OR: 0.79, 95% CI: 0.63 to 0.98). However, there was no significant difference in the live birth rate for the CAL protocol (**Figure 6**, OR: 0.85, 95% CI: 0.64 to 1.14).

#### 3.3.6 Cumulative live birth rate

**Figure 7:**
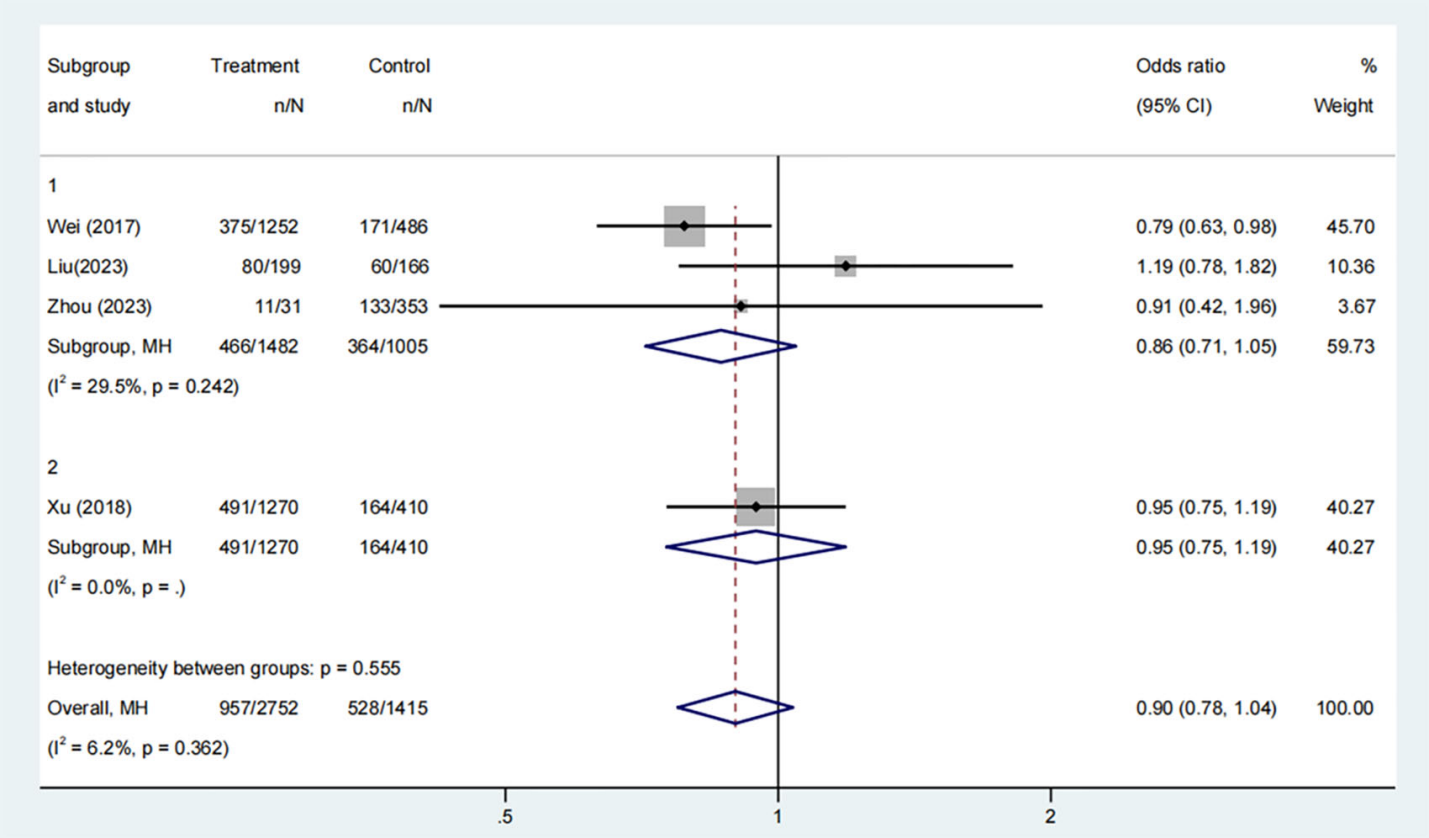
Analysis of cumulative live birth rate, including the comparison between different ovulation induction regimens.

Four studies, involving 2682 participants, examined the cumulative live birth rates in groups that received COC pre-treatment and control groups. The meta-analysis found no significant difference between the groups (**Figure 7**, OR: 0.90, 95% CI: 0.78 to 1.04). Subgroup analysis for both COC pre-treatment protocols, CAA (**Figure 7**, OR: 0.86, 95% CI: 0.71 to 1.05) and CAL (**Figure 7**, OR: 0.95, 95% CI: 0.75 to 1.19), showed no significant difference compared to the control group.

#### 3.3.7 OHSS

**Figure 8:**
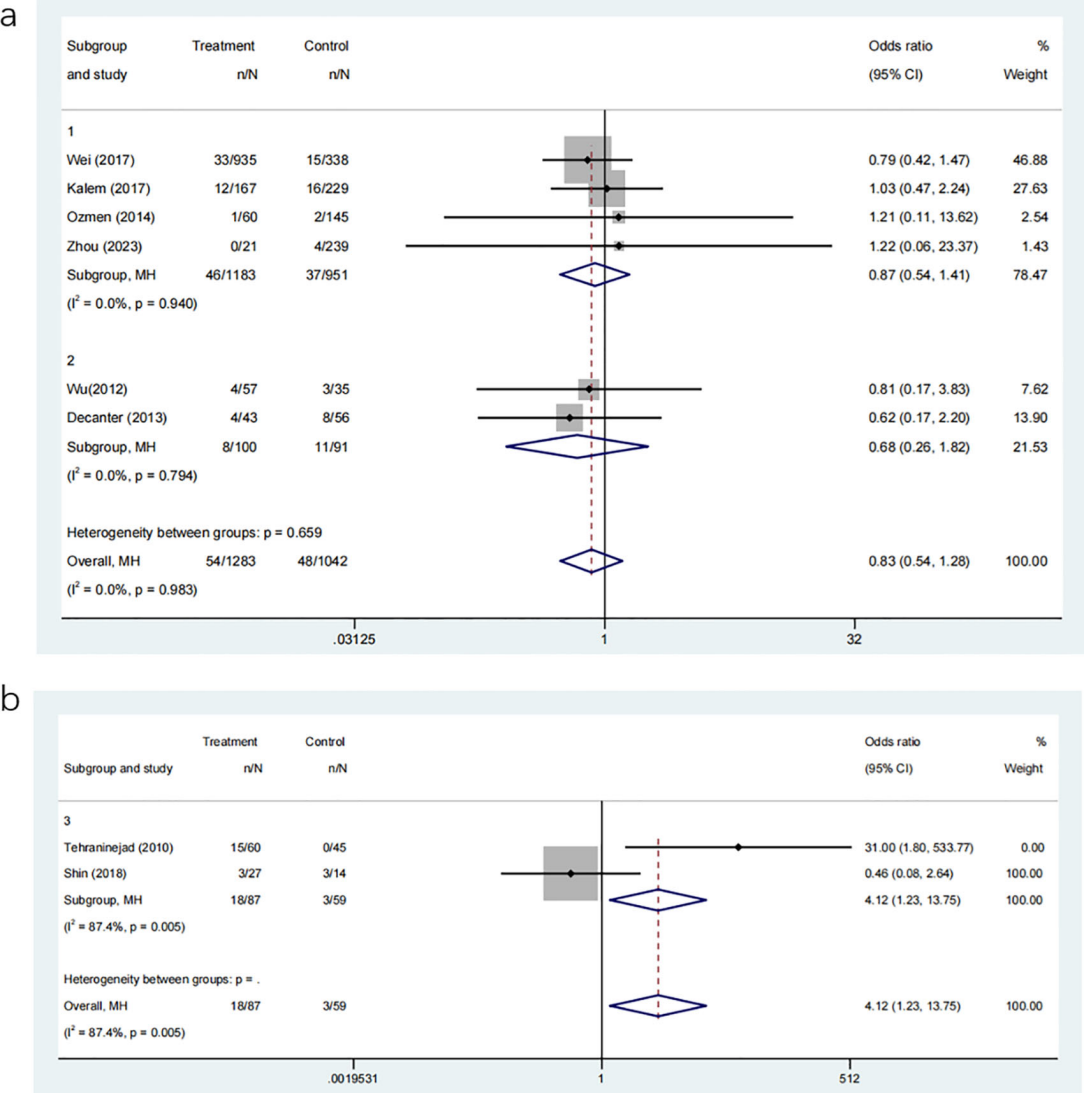
**(a)** Analysis of the OHSS,including the comparison between different ovulation induction regimens; **(b)** Analysis of OHSS after COC preconditioning with different ovulation promoting schemes.

Six studies, involving a total of 2223 participants, examined the occurrence of OHSS in groups that received COC pre-treatment compared to control groups. The analysis indicated no significant increase in the risk of OHSS in the COC pre-treatment group compared to the control group (**Figure 8a**: OR: 0.83, 95% CI: 0.54 to 1.28). Subgroup analyses for different COC pre-treatment protocols revealed no significant advantage in reducing OHSS incidence for both CAA (**Figure 8a**: OR: 0.87, 95% CI: 0.54 to 1.41) and CAL protocols (**Figure 8a**: OR: 0.68, 95% CI: 0.26 to 1.82) compared to the control group without COC pre-treatment.

Two further investigations, involving 125 participants in total, directly examined the incidence of OHSS between the CAA and CAL COC pre-treatment procedures. The findings revealed that the CAL group had a significantly greater occurrence rate of OHSS compared to the CAA group (**Figure 8b**: OR: 4.12, 95% CI: 1.23 to 13.75), indicating that the COC pre-treatment methods have varying effects on the risk of OHSS.

#### 3.3.8 Gonadotropin dosage

**Figure 9:**
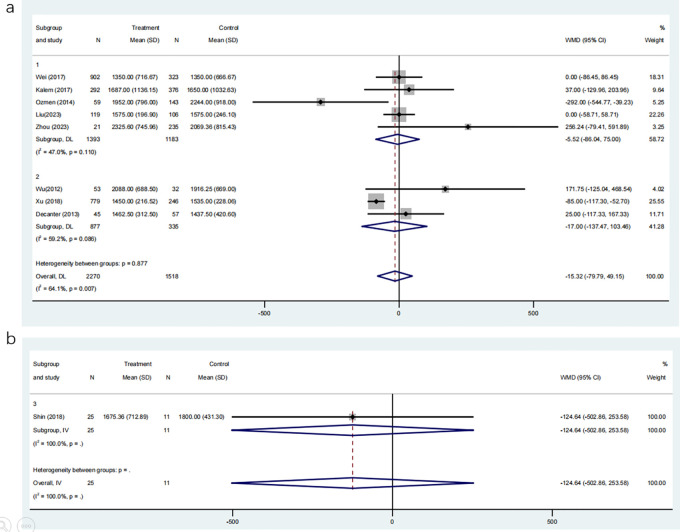
**(a)** Analysis of the Gonadotropin Dosage,including the comparison between different ovulation induction regimens; **(b)** Analysis of Gonadotropin Dosage after COC preconditioning with different ovulation promoting schemes.

Eight studies, involving 3788 participants, compared the required dosage of gonadotropin between COC pre-treatment groups and control groups. The meta-analysis demonstrated no significant difference in gonadotropin dosage between the two groups (**Figure 9a**; MD: -15.32, 95% CI: -79.79 to 49.15).

Subgroup analyses based on ovulation induction protocols showed similar results: the CAA group (MD: -5.52, 95% CI: -86.04 to 75.00) and the CAL group (MD: -17.00, 95% CI: -137.47 to 103.46) did not significantly differ from their respective control groups without COC pre-treatment. Additionally, a direct comparison of gonadotropin dosage between the CAA and CAL groups, involving 36 participants, revealed no significant difference (**Figure 9b**; MD: -124.64, 95% CI: -502.86 to 253.58). These findings suggest that COC pre-treatment does not significantly affect the amount of gonadotropin required for controlled ovarian stimulation, regardless of the subsequent ovulation induction protocol.

## 4 Discussion

ART is an effective clinical treatment for PCOS complicated with infertility, although in clinical practice, PCOS In patients with infertility, oral COC is used to regulate the body prior to ovulation induction, thereby increasing LH and testosterone (T) levels, which can promote ovulation and may reduce miscarriage rates (13). However, it is unclear whether oral COC prior to ART treatment in patients with PCOS and infertility improves clinical pregnancy rates, live birth rates, and reduces the incidence of miscarriage and OHSS (26, 31).Based on the above questions, this paper conducted a meta-analysis of 11 articles in the final inclusion area through a systematic search database, and found that COC pre-treatment did not affect the implantation rate, pregnancy rate, cumulative live birth rate and OHSS incidence rate, and there was no statistical difference in the above indicators. However, the miscarriage rate in the COC pre-treatment group was significantly higher than that in the control group and the live birth rate was lower than that in the control group, and the risk of miscarriage in the COC pre-treatment group with the antagonist regimen was higher than that in the control group, and there was no difference in the miscarriage rate with the GnRH agonist (standard long-term regimen) with COC pre-treatment compared with the control group. Subcomponent analysis showed that the incidence of OHSS with GnRH agonists (standard long-term regimens) after COC pre-treatment was significantly higher than that with antagonists after COC pre-treatment.

Our meta-analysis revealed that COC conditioning did not have a significant impact on pregnancy rates, which is in direct contradiction to previous research. Based on previous literature, although oral contraceptives can block luteinizing hormone and reduce progesterone synthesis can help prevent the negative impact of progesterone on the endometrium’s ability to accept embryos, thereby increasing the implantation rate (32), COC pre-treatment can also cause some adverse clinical outcomes, mainly including the following two aspects:1.Contraceptive pill pre-treatment can affect endometrial receptivity and embryo quality, which in turn affects embryo implantation and reduces clinical pregnancy rates (33, 34); 2.COC pre-treatment reduces the sensitivity of the ovaries to LH and can lead to a decrease in follicular quality (35).Furthermore, the reduction in the thickness of the endometrial lining can impact the spiral artery located near the basal layer of embryonic implantation. This, in turn, leads to the generation of reactive oxygen species, which create elevated levels of oxygen that impede the development of the embryo (36). As a result, the miscarriage rate was increased after COC pre-treatment, which is consistent with our findings. Although the birth control pill does not directly lead to a decrease in the live birth rate (28), it is currently suggested that lower endogenous LH levels after treatment with the birth control pill may impair oocyte capacity or endometrial receptivity, resulting in a decrease in the live birth rate (37). In addition, individual variations in serum hormone responses to COC pre-treatment may influence clinical outcomes. Specifically, COC-induced suppression of LH levels and modulation of estradiol concentrations could impact oocyte maturation, follicular development, and endometrial receptivity. Over-suppression of LH might impair folliculogenesis and reduce oocyte competence, whereas suboptimal estrogen priming could compromise endometrial thickness and receptivity, collectively leading to variations in implantation success, pregnancy maintenance, and miscarriage risk among PCOS patients undergoing ART (38).

However, in terms of cumulative live birth rates, a total of 4 studies involving 2682 patients were included, and there was no difference between the COC pre-treatment and control groups, and there was no difference in the cumulative live birth rate between the COC pre-treatment and different ovulation induction regimens after COC pre-treatment, and there was no difference between the cumulative live birth rate and the non-COC pre-treatment, which is different from previous reports (39). One important factor potentially contributing to these findings is the variation in embryo transplantation strategies across studies. Fresh embryo transfer (ET) and frozen embryo transfer (FET) differ in their impact on endometrial receptivity and pregnancy outcomes. Previous studies have consistently shown that FET is associated with higher implantation and live birth rates compared to fresh ET, possibly due to better endometrial synchronization and a more favorable intrauterine environment (23, 28, 40). Fresh ET cycles may lead to supraphysiological estrogen levels and altered vascular endothelial growth factor (VEGF) profiles, resulting in impaired endometrial receptivity and a lower cumulative live birth rate (41). Furthermore, the impact of COC pre-treatment appears diminished during FET cycles, as its prolonged effect on endometrial development is minimized (23). Since the included studies involved both fresh and frozen transfers, without uniform stratification, differences in transplantation strategy could have contributed to clinical heterogeneity and influenced the observed results. Another reason is that considering that contraceptive pill pre-treatment affects the receptivity of the endometrium and damages oocytes and leads to a decrease in the live birth rate, compared with the previous study, two new studies were included in the statistics of the cumulative live birth rate, and 140 cases of COC pre-treatment were added, while there were 341 cases in the control group, and the ovulation induction regimen and transplantation strategy could not be compared, and the difference in the number of cases was currently considered first.

The incidence of OHSS is a clinically undesirable outcome of concern. Oral contraceptive pre-treatment is often used to reduce the incidence of OHSS during assisted reproductive superovulation induction (42). Individuals diagnosed with polycystic ovary syndrome have a notably higher likelihood of developing ovarian hyperstimulation syndrome as a result of polycystic manifestations. However, in our article, there was no significant difference in the incidence of OHSS between the COC pre-treatment and the control group, but the incidence of OHSS with COC pre-treatment and with GnRH agonists (standard long-term regimens) was higher than that of antagonists, indicating that the effect of COC pre-treatment methods on OHSS risk was different, which is consistent with the reports (22, 43), and it is generally believed that GnRH antagonists inhibit the pituitary gland by directly competing with endogenous GnRH, As a result, the lower gonadotropin dose requirement and lower peak E2 levels in the GnRH antagonist regimen lead to a lower probability of OHSS, although we found no significant difference in gonadotropin dose between the contraceptive conditioning and control groups, and there was no significant difference in gonadotropin dose between the two ovulation induction regimens after contraceptive conditioning (44). Therefore, low E2 peaks are not the only cause of OHSS, and further research is needed (21). Additionally, for the direct comparison of OHSS incidence between the CAA and CAL COC pre-treatment groups, 125 participants were analyzed. These participants were not independent from the main analysis cohort but were derived from subgroup data within two of the six selected studies included in the OHSS analysis. Subgroup analysis revealed that the CAL group had a significantly higher OHSS rate compared to the CAA group. However, given the relatively small sample size in this direct comparison, the robustness of this finding may be limited, and caution is warranted when interpreting these results.

Simultaneously, we have identified specific constraints in this study. The study lacked complete uniformity in both the type of COC used and the authors’ criteria for diagnosing PCOS. Second, in the ovulation induction regimen, Pan used GnRH agonist (standard long regimen), GnRHa short regimen and antagonist regimen supplement, but the specific number could not be distinguished in the study, all GnRH agonists (standard long regimen) were used as statistics, Shin’s report included early antagonist regimen and conventional antagonist regimen, and when statistically stating the differences between different regimens, both considered GnRH antagonists to be analyzed, and Zhuo’s report was divided into low LH group and high LH group without COC pre-treatment, and they were all classified as non-COC pre-treatment groups in statistics. Third, there are many causes of infertility in PCOS patients, but due to the lack of relevant data, it is impossible to independently analyze the different causes of infertility. Fourth, while we performed subgroup analyses based on ovulation induction regimens to address some of the heterogeneity in ART technologies, variations in transplantation strategies (e.g., fresh versus frozen embryo transfer) and laboratory techniques (e.g., IVF versus ICSI) across the included studies could still influence the outcomes. Due to the limited number of studies and insufficient data, we were unable to conduct further subgroup analyses for these factors, which may introduce residual confounding. Fifth, the analysis of OHSS incidence comparing COC pre-treatment with GnRH agonist (CAL) versus GnRH antagonist (CAA) protocols relied on two additional studies with a total of 125 participants, a relatively small sample size that may limit the robustness of the findings. Additionally, direct comparisons of OHSS rates between CAA and CAL within the six primary studies were not feasible, as most studies utilized a single ovulation induction regimen, precluding such analyses. Sixth, the included studies used various COC formulations (e.g., ethinylestradiol combined with desogestrel, drospirenone, or cyproterone acetate), but lacked consistent reporting of dosage and treatment duration, precluding stratified analysis. Thus, the influence of COC regimen characteristics on clinical outcomes remains unclear. However, gonadotropin dosage did not differ significantly between COC pre-treated and control groups, suggesting that COC pre-treatment did not substantially affect gonadotropin requirements. Future studies should clarify the dose-response effects of COC pre-treatment on ART outcomes in PCOS patients.

## 5 Conclusion

Administering COC pre-treatment to patients with PCOS and infertility prior to ART treatment does not impact the implantation rate, pregnancy rate, cumulative live birth rate, and OHSS incidence. However, it does lead to an increase in the miscarriage rate. Therefore, oral COC contraceptives are not recommended for patients with PCOS and infertility prior to ART treatment. However, more research is needed to validate this.

## Data Availability

The original contributions presented in the study are included in the article/**Supplementary Material**. Further inquiries can be directed to the corresponding author.
