# Supplementary material for: Effect of pre-treatment with oral short-acting contraceptives on assisted reproductive technology outcomes in patients with polycystic ovary syndrome: a meta-analysis
Source: Front Endocrinol (Lausanne). 2025 Jun 5;16:1545508. doi: 10.3389/fendo.2025.1545508 (PMC12176574; doi:10.3389/fendo.2025.1545508)
Supplement: Supplementary file 1 [file Table1.docx]

**Supplementary**

Table of Contents:

[Supplementary 1: Search Strategy 2](#_Toc159076731)

[Supplementary 2: Risk of Bias](#_Toc159076747) 3

# Supplementary 1: Search Strategy

***Search Strategy (coverage from inception to 6 January 2025):***

| #1 | Search: Polycystic Ovary Syndrome[MeSH Terms] |
| --- | --- |
| #2 | Search: “polycystic ovary syndrome”[Title/Abstract] OR “PCOS”[Title/Abstract] OR “Stein-Leventhal syndrome”[Title/Abstract] OR “sclerocystic ovary”[Title/Abstract] OR “sclerocystic ovaries”[Title/Abstract] |
| #3 | Search: #1 OR #2 |
| #4 | Search: **Contraceptives, Oral, Combined[MeSH Terms]** |
| #5 | Search: **Oral Contraceptives, Low-Dose[MeSH Terms]** |
| #6 | Search: “combined oral contraceptive*”[Title/Abstract] OR “oral contraceptive pill”[Title/Abstract] OR “oral contraceptive*”[Title/Abstract] OR “short-acting oral contraceptive*”[Title/Abstract] OR “low-dose oral contraceptive*”[Title/Abstract] |
| #7 | Search: #4 OR #5 OR #6 |
| #8 | Search: Reproductive Techniques, Assisted[MeSH Terms] |
| #9 | Search: “assisted reproductive technolog*”[Title/Abstract] OR “ART”[Title/Abstract] OR “in vitro fertilization”[Title/Abstract] OR “IVF”[Title/Abstract] OR “intracytoplasmic sperm injection”[Title/Abstract] OR “ICSI”[Title/Abstract] |
| #10 | Search: #8 OR #9 |
| #11 | Search: #3 AND #7 AND #10 |
| #12 | Search: randomized controlled trial[pt] OR controlled clinical trial[pt] OR randomized[tiab] OR prospectiv*[tiab] OR cohort[tiab] OR case-control[tiab] OR observational[tiab] |
| #13 | Search: #11 AND #12 NOT (animals[mh] NOT humans[mh]) |

# Supplementary 2: Risk of Bias

Table 2.1 Risk of Bias Assessment for RCTs (RoB 2 tool)

| **Study** | **Bias arising from the randomization process** | **Bias due to deviations from intended intervention** | **Bias due to missing outcome data** | **Bias in measurement of the outcome** | **Bias in selection of the reported result** | **Overall** |
| --- | --- | --- | --- | --- | --- | --- |
| Tehraninejad (2010) | Low Risk | Low Risk | Low Risk | Low Risk | Low Risk | Low Risk |
| Shin (2018) | Low Risk | Low Risk | Low Risk | Low Risk | Low Risk | Low Risk |

Table 2.2 Risk of Bias Assessment for Non-RCTs (ROBINS-I tool)

| **Study** | **Bias due to Confounding** | **Bias in Selection of Participants** | **Bias in Classification of Interventions** | **Bias due to Deviations from Intended Interventions** | **Bias due to Missing Data** | **Bias in Measurement of Outcomes** | **Bias in Selection of the Reported Result** | **Overall Bias Judgment** |
| --- | --- | --- | --- | --- | --- | --- | --- | --- |
| Wei (2017) | Moderate | Low | Low | Low | Low | Low | Low | Moderate |
| Pan (2014) | Serious | Moderate | Low | Low | Moderate | Low | Moderate | Serious |
| Kalem (2017) | Moderate | Low | Low | Low | Low | Low | Low | Moderate |
| Ozmen (2014) | Moderate | Low | Low | Low | Low | Low | Low | Moderate |
| Wu (2012) | Moderate | Low | Low | Low | Low | Low | Low | Moderate |
| Xu (2019) | Serious | Moderate | Low | Low | Moderate | Low | Moderate | Serious |
| Decanter (2013) | Moderate | Low | Low | Low | Low | Low | Low | Moderate |
| Liu (2023) | Moderate | Low | Low | Low | Low | Low | Low | Moderate |
| Zhou (2023) | Moderate | Low | Low | Low | Low | Low | Low | Moderate |
